# A critical review of cannabis in medicine and dentistry: A look back and the path forward

**DOI:** 10.1002/cre2.564

**Published:** 2022-04-01

**Authors:** Ammaar H. Abidi, Sahar S. Alghamdi, Karen Derefinko

**Affiliations:** ^1^ College of Dentistry, Department of Bioscience Research The University of Tennessee Health Science Center Memphis Tennessee USA; ^2^ College of Dentistry, Department of General Dentistry The University of Tennessee Health Science Center Memphis Tennessee USA; ^3^ Department of Phamaceutical Sciences, College of Pharmacy King Saud Bin Abdulaziz University for Health Sciences Riyadh Saudi Arabia; ^4^ King Abdullah International Medical Research Center (KAIMRC) Ministry of National Guard Health Affairs Riyadh Kingdom of Saudi Arabia; ^5^ College of Medicine, Department of Pharmacology, Addiction Science, and Toxicology The University of Tennessee Health Science Center Memphis Tennessee USA; ^6^ College of Medicine, Department of Preventive Medicine The University of Tennessee Health Science Center Memphis Tennessee USA

**Keywords:** cannabinoids, dentistry, pain, pharmacotherapy

## Abstract

**Introduction:**

In the last two decades, our understanding of the therapeutic utility and medicinal properties of cannabis has greatly changed. This change has been accompanied by widespread cannabis use in various communities and different age groups, especially within the United States. With this increase, we should consider the potential effects of cannabis–hemp on general public health and how they could alter therapeutic outcomes.

**Material and Methods:**

The present investigation examined cannabis use for recreational and therapeutic use and a review of pertinent indexed literature was performed. The focused question evaluates “how cannabis or hemp products impact health parameters and do they provide potential therapeutic value in dentistry, and how do they interact with conventional medicines (drugs).” Indexed databases (PubMed/Medline, EMBASE) were searched without any time restrictions but language was restricted to English.

**Results:**

The review highlights dental concerns of cannabis usage, the need to understand the endocannabinoid system (ECS), cannabinoid receptor system, its endogenous ligands, pharmacology, metabolism, current oral health, and medical dilemma to ascertain the detrimental or beneficial effects of using cannabis–hemp products. The pharmacological effects of pure cannabidiol (CBD) have been studied extensively while cannabis extracts can vary significantly and lack empirical studies. Several metabolic pathways are affected by cannabis use and could pose a potential drug interaction. The chronic use of cannabis is associated with health issues, but the therapeutic potential is multifold since there is a regulatory role of ECS in many pathologies.

**Conclusion:**

Current shortcomings in understanding the benefits of cannabis or hemp products are limited due to pharmacological and clinical effects not being predictable, while marketed products vary greatly in phytocompounds warrant further empirical investigation. Given the healthcare challenges to manage acute and chronic pain, this review highlights both cannabis and CBD‐hemp extracts to help identify the therapeutic application for patient populations suffering from anxiety, inflammation, and dental pain.

## 1 THE HISTORICAL USE OF CANNABIS

The properties of the marijuana plant (*Cannabis sativa*) are well documented for its use in textile manufacture, health benefits, and healing properties. The ancient world shows several medical records describing the usage of cannabis as a therapeutic agent by several cultures (Mikuriya, 1969). About 2000 years ago, the Chinese culture used cannabis to gain balance and harmony between Yin and Yang that prevent disease formation. The plant seeds were utilized as a laxative for treating constipation with the advantage that cannabis seeds lack the main active constituent Δ^9^‐tetrahydrocannabinol (Δ^9^‐THC) but are rich in fatty acids. These fatty acids such as *γ*‐linoleic acid were applied topically to treat several skin conditions and orally for the treatment of atherosclerosis, osteoporosis, and inflammatory‐based conditions. In India, the psychoactive effect of cannabis was popular, which supported its broad use with religious and meditation rituals. Moreover, ayurvedic (Hindu/Indian) remedies were used for their analgesic, hypnotic, and antispasmodic properties (Mechoulam & Carlini, 1978). The wide use of cannabis in India has resulted in the spreading of the plant to Arabia for medicinal purposes. It has been reported in Ibn al Badri's treatise of Hashish, in which Chamberlain of the Caliphate Council's son in Baghdad, Zahir‐ad‐din was cured of epilepsy but became dependent on it and used it for the rest of his life (Lozano, 1997; Mechoulam, 1986).

The western world, however, saw the use of cannabis for its medicinal value and recreation usage years later. For much of the 1800s and early 1900s, the active ingredients in cannabis were unknown and it was introduced later by O'Shaughnessy (an Irish physician) and Moreau (a French psychiatrist) in the second half of the 19th century (Aldrich, 1997; Mechoulam & Carlini, 1978). The first scientific conference that discussed cannabis therapeutics was held in Ohio, United States in 1860 (McMeens, 1860), and the first characterization of cannabinol (CBN) was done in 1932 (Cahn, 1932) followed by the chemical synthesis of CBN and cannabidiol (CBD) in 1940 (Adams et al., 1940; Jacob & Todd, 1940). In 1964, the psychoactive constituent of cannabis plant Δ^9^‐THC was isolated and partially synthesized (Gaoni & Mechoulam, 1964). Devane et al. (1988) provided evidence for the presence of specific cannabinoid receptors in the rat brain and confirmed the isolation of cannabinoid receptor type 1 (CB1R). Shortly after, Munro et al. (1993) isolated and cloned the second cannabinoid receptor subtype, cannabinoid receptor type 2 (CB2R) in a human promyelocytic cell line (HL60).

In the early 20th century, more than thousands of publications studied the medicinal properties and therapeutic applications of cannabis in multiple disease conditions. Moreover, with the widespread cannabis use in various communities and age groups, healthcare practitioners should be cautious about cannabis's effects on general public health and how it can affect several diseases. Interestingly, with regard to dental health, it has been reported that marijuana users exhibit more decay, poor oral hygiene, and greater plaque index than noncannabis users. There are also additional intraoral conditions reported with cannabis users that include tongue carcinoma, gingival hyperplasia, xerostomia, uvulitis, and fiery red gingivitis (for excellent reviews see Almadori et al., 1990; Darling & Arendorf, 1992; Versteeg et al., 2008). Therefore, this review will shed light on the cannabinoid receptor system, its endogenous ligands, pharmacology, metabolism, and the current medical/dental dilemma.

## 2 MATERIALS AND METHODS

### 2.1 Ethical guidelines

The present study is a review and no patients have been involved in the present study, ethical considerations or protection of human subjects and animals were noted by the Helsinki Declaration of 1975, as revised in 2013. Therefore, the protocol was exempted from prior ethical approval from an institutional review board (IRB).

### 2.2 Search strategy and inclusion

Indexed databases (PubMed/Medline [National Library of Medicine]), EMBASE were independently searched without time but were restricted to the English language.

The focused question evaluates “how cannabis or hemp products impact health parameters and do they provide potential therapeutic value in dentistry, and how do they interact with conventional medicines (drugs).”

Original clinical studies, original studies on humans, animal models, in/ex‐vivo cell studies, case reports/series, letters to the Editor, reviews, perspectives, and commentaries were all sought and were eligible for inclusion. The exclusion criteria included conference papers/proceedings.

### 2.3 Databases search protocol and keywords

Language restrictions using different combinations of the following free text keywords: cannabis OR “cannabinoids” OR “phytocannabinoids” AND “marijuana” OR “hemp” AND “nociception” AND “inflammation” AND “anxiety” AND “dental pain” OR “acute pain” OR “chronic pain” AND “dentistry.”

### 2.4 Data extraction

Titles and abstracts obtained from the initial search were screened and any publications that failed to abide by the inclusion criteria were excluded. Disagreements related to literature or improper methodology were discussed and consulted by the authors.

For all studies that fulfilled the eligibility criteria, data extraction comprised of the following parameters: (1) author and year, (2) subjects and their characteristics, (3) study groups, (4) route of administration, (5) concentration and frequency of cannabinoids (6) Inflammation, nociception, pain, analgesia (7) main results and clinical outcome (8) conclusion and analyses.

## 3 THE ENDOCANNABINOID SYSTEM (ECS)

The CB1R was initially identified in 1988 in rat brain tissue using tritiated nonselective agonist [^3^H]‐CP‐55,940 (Devane et al., 1988) followed by cloning cDNA of CB1R from rat cerebral cortex in 1990 (Matsuda et al., 1990). The following year, human CB1R was isolated from a human brain stem, and it was identified to share 97.3% homology with rat CB1R (Gérard et al., 1991). Using [^3^H] CP 55,940, it was found that CB1R is mainly condensed in the brain regions, such as basal ganglia, substantia nigra, hippocampus, and cerebellum (Herkenham et al., 1990). Moreover, it was reported by several studies that CB1R demonstrated similar distribution between species, such as the rat, monkey, dog, and pig with minor differences (Herkenham et al., 1990; Mailleux & Vanderhaeghen, 1992; Pertwee, 1997). Additionally, the CB1R has been documented to be expressed in some peripheral tissues at a lower level, such as ileum longitudinal smooth muscles (Croci et al., 1998) and cardiovascular tissues (Szabo et al., 2001; Wagner et al., 2001). The wide distribution of CB1R in the human body suggests a crucial regulatory role in glucose metabolism (Nogueiras et al., 2009), food intake (Ravinet Trillou et al., 2004), neurotransmitter release modulation (through M‐type potassium channels) (D. J. Kim & Thayer, 2000; Schweitzer, 2000) cancer (Pisanti et al., 2013) and, more importantly, pain management (Pertwee, 2001).

In 1993, the CB2R was discovered and characterized using a human promyelocytic leukemic cell line (HL60). Initially, CB2R was named as a peripheral cannabinoid receptor because of the high expression in peripheral immune cells, such as spleen's macrophages (Munro et al., 1993). B‐cells, natural killer cells, monocytes, neutrophils, and T cells (Galiègue et al., 1995). However, recent advances in research tools and techniques led to the identification of CB2R in the brain microglia during inflammatory conditions (Kearn & Hilliard, 1997). The amino acid residues in the CB2R structure showed more diversity in the C‐terminus region among different species (human, rat, mouse) (Brown et al., 2002; Griffin et al., 2000). The limited distribution of CB2R in the immune cells suggests that this receptor plays an important regulatory role in inflammation and serves as a novel target for multiple neuroinflammatory disease conditions, such as Alzheimer's disease (AD), Parkinson's disease (PD), and multiple sclerosis (MS) (Mecha et al., 2016).

**Figure 1:**
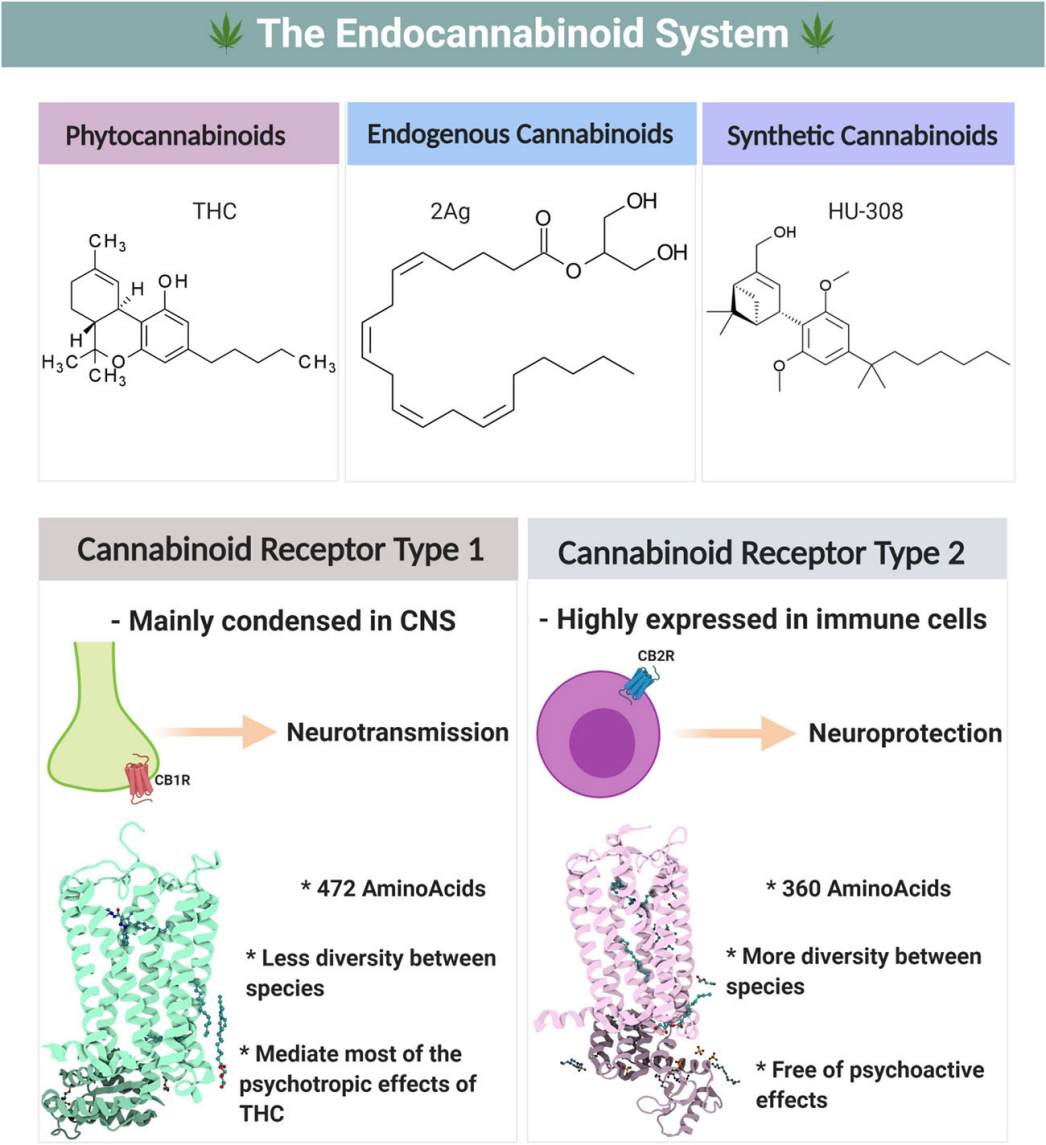
The three main categories of cannabinoid ligands with the major differences between cannabinoid receptor type 1 and type 2 (CB1 and CB2) receptors. Both cannabinoid receptors are found throughout the body; however, tissue specificity does exist with CB1R: Mainly CNS and CB2R: Mainly immune cells. The pharmacological activity of the CBRs is mediated via G‐protein‐coupled receptors, specifically G_αi_, which modulate the adenylyl cyclase (AC) activity and the production of the intracellular cAMP. Created with BioRender.com

The two cannabinoid receptors belong to the superfamily of G‐protein‐coupled receptors (GPCRs—Class A, Figure 1) that have seven transmembrane alpha‐helices with a glycosylated *N*‐terminus and intracellular C‐terminus (Bramblett et al., 1995). The homology between the CB1/2 receptors is approximately 44% in the amino acid sequence and 68% in the ligand‐binding site (Munro et al., 1993). The first endogenous ligand for the cannabinoid receptors was discovered from the porcine brain two years after the discovery of CB1R and named arachidonoyl ethanolamide (anandamide, AEA). The binding studies of AEA demonstrated a high binding affinity toward CB1R and it also exhibited similar psychoactive properties of Δ^9^‐THC when tested in rodents (Devane et al., 1992). In 1995, the second endogenous cannabinoid ligand 2‐arachidonoylglycerol (2‐AG) was discovered and isolated from the canine gut (Mechoulam et al., 1995) and both ligands are lipids analogs that are synthesized on‐demand after physiological or pathogen stimuli.

## 4 THE PHARMACOLOGY OF CANNABINOID LIGANDS

### 4.1 Downstream signaling

The complexity of signaling pathways shows tissue specificity and may have distinctly different molecular signaling (Cabral & Griffin‐Thomas, 2009), resulting in a variety of processes being affected by the CB receptors. The canonical cannabinoid signaling pathway regulates multiple downstream effectors, including cyclic adenosine monophosphate (cAMP), ion channels, and mitogen‐activated protein kinase (MAPKs). CB1/2 receptors couple with the heterotrimeric G‐protein complex via pertussis toxin (PTX) sensitive G_α__i_ that is present in most cell types ranging from neuronal to immune cells (Felder et al., 1992; Howlett et al., 1986). The ion channel modulation can occur as a result of G_α__i_ activation and G_βγ_ subunits. At presynaptic terminals, CB1R (via G_βγ_) activates A‐type potassium (K^+^) channels, which increases K^+^ influx and inhibits L, N, P, Q type Ca^2+^ channels that decrease the calcium concentration inside the cell, which plays a crucial role in neurotransmitters release (Caulfield & Brown, 1992; Mackie et al., 1995). The CB1/2 receptors are upstream regulators of MAPK activation, which regulate a wide variety of cell functions, such as cell growth, differentiation, and apoptosis, reviewed by Seger and Krebs (1995) CB1R positively regulates the extracellular signal‐regulated kinase 1 or 2 (ERK_1/2_) via G_α__i_ protein (Daigle et al., 2008) and β‐arrestin pathways (Ahn et al., 2013). Moreover, several cannabinoid ligands activated ERK_1/2_ via CB2R, suggesting the ability of CB2R to mediate MAPKs activation (Atwood et al., 2012).

The immune‐modulatory signaling pathways of CB2 receptors regulate cAMP, MAPK, modulation of intracellular calcium (Demuth & Molleman, 2006), and other signaling molecules, including transcriptional factors, such as nuclear factor‐κB (NF‐κB) (Jüttler et al., 2004; Toguri et al., 2014). Nuclear factor of activated T‐cells (NFAT) (Kaplan & Kaminski, 2007; Kaplan et al., 2008) and activator protein 1 (AP‐1) (Toguri et al., 2014). CB2 receptor agonists decrease intracellular cAMP (Yao & Mackie, 2009), while inverse agonists increase intracellular cAMP. Therefore, CB2R ligands can potentially mediate phosphorylation of cAMP response element (CRE)‐binding protein (p‐CREB) to activate CRE that would lead to anti‐inflammatory responses (Börner et al., 2009; Bu et al., 2016; Montecucco et al., 2008; Wen et al., 2010) and direct inhibition of NF‐κB complex by diminishing cAMP binding protein (CBP) availability, which is a coactivator for the binding of the NF‐κB complex (Wen et al., 2010).

### 4.2 Endogenous cannabinoids

The first discovered endocannabinoid ligand AEA was classified as a fatty acid amide that behaves as a nonselective partial agonist at CBRs (Devane et al., 1992; Felder et al., 1996). This ligand binds with greater affinity to CB1R (*K*_i_ = 71.7 nM) and has been reported to behave as a partial and full agonist at CB1R, depending on tissues and functional assays used. At CB2R, AEA binds with lower affinity (*K*_i_ = 279 nM) and demonstrated partial agonist or antagonist pharmacological profiles, depending on the experimental method that was applied (Gonsiorek et al., 2000). In neurons, AEA is synthesized on demand (in response to stimuli or infection) via phospholipid‐dependent pathway that utilizes *N*‐arachidonyl phosphatidylethanolamine (*N*‐arachidonyl PE) as a starting precursor. The cleavage of *N*‐arachidonyl PE into AEA is catalyzed by an enzyme named phospholipase D (PLD), which produces two main products, AEA and phosphatidic acid (intermediate metabolite). Following AEA released into the extracellular space, it binds and activates cannabinoid receptors to produce a variety of biological responses. To prevent depletion of the main precursor *N*‐arachidonyl PE, an enzyme called *N*‐acyl transferase enzyme (NAT) is responsible for removing arachidonic acid moiety from phosphatidylcholine (PC) and reattaching it to the primary amino group in PE. The hydrolysis of AEA is mediated by fatty acid amide hydrolase (FAAH) or AEA amidohydrolase (AAH), which is highly expressed in the brain and liver. Two main products resulting from AEA hydrolysis are arachidonic acid and ethanolamine. Arachidonic acid can feed into the eicosanoid pathway (van Kranen & Siezen, 2016) or is further utilized and incorporated into phospholipids to generate PC that can be subsequently used in PE formation, reviewed by  Pacher et al. (2006).

Moreover, 2‐AG was the second endocannabinoid identified and it demonstrated full agonist activity at CB1/2 receptors, with a lower affinity toward CB1R (K_i_ = 472 nM) but full efficacy compared to AEA. The affinity of 2‐AG for CB2R was lower with a *K_i_* value of 1.4 μM in transfected cells and comparable values were reported in brain tissues (Sugiura et al., 1995). Unlike AEA, 2‐AG has two main biosynthetic pathways. The first pathway involves hydrolysis of phosphatidylinositol 4, 5 bisphosphate (PIP2) into diacylglycerol (DAG) and inositol 1, 4, 5 triphosphates (I_3_P). This rapid hydrolysis is mediated by phospholipase C (PLC β or γ), which uses Ca^2+^ as a cofactor. DAG is further hydrolyzed to yield 2‐AG, which is carried out by DAG lipase (α or β) enthe zyme. The second biosynthetic pathway is through the conversion of phosphatidylinositol 4, 5 bisphosphates (PIP_2_) into 2‐arachidonoylglycerol lysophosphatidic acid (2AG‐LPI), which is mediated by phospholipase A1 enzyme (PLA1). The resulting 2‐AG‐LPI intermediate is further hydrolyzed by specific PLC 5 (lyso‐PLC) to give 2‐AG. The degradation of 2‐AG is mediated by uncharacterized monoacylglycerol lipase (MAGL) that is highly expressed in brain neurons. The intracellular hydrolysis of 2‐AG produces two main products, arachidonic acid, and glycerol, which can be further recycled and utilized, as reviewed by Piomelli et al. (2000). It is important to highlight the lipid pathways activated by ECS, which are a highly regulated and complex lipid network that exhibit crosstalk between eicosanoids and endocannabinoids (Pertwee, 2006), modulating essential pathways involved with a variety of host responses.

The circulating endocannabinoids come from multiple organ systems and serve to regulate many homeostatic functions. The endocannabinoid concentration can be released and inactivated in the blood (Hillard, 2018). Similarly, the upregulation of AEA after periodontal surgery reveals a protective mechanism that supports the wound healing process and promotes the proliferation of the gingival fibroblasts (Kozono et al., 2010). Other studies examining the dental application of topical methanandamide, a stable analog of AEA, showed a significant reduction in alveolar bone loss in the rat model of periodontitis (PD) (Ossola et al., 2012). Therefore, AEA or 2‐AG involvement in gingivitis and PD along with the systemic role of ECS in immunomodulation seems to play a major role. During inflammation, the CB2R are upregulated (Turcotte et al., 2016) and in chronic inflammatory disease like PD, the efficacy of endocannabinoids, that is, 2‐AG or AEA in activating the ECS may be insufficient to overcome the proinflammatory feedback loop (dysregulation) (Abidi et al., 2020). The limitations of the endogenous ligands may be due to short half‐life, limited efficacy, and reduced potency in chronic inflammation. Ongoing studies are trying to overcome the insufficiencies of the endogenous system by utilizing the phytocannabinoids or synthetic ligands to sustain the activation of the ECS and provide long‐term proresolution/anti‐inflammatory effects in chronic inflammatory conditions.

## 5 PHYTOCANNABINOIDS

The female plant of *C. sativa* contains hundreds of various lipid‐soluble compounds that share CBRs as molecular targets. The two molecules Δ^9^‐THC and CBD are the most studied pCBs in literature. At CB1R, the affinity of Δ^9^‐THC is ranging from 5 to 80.3 nM and it demonstrated a comparable low nanomolar range affinity (*K_i_* = 3–75 nM) at CB2R. Additionally, it has been reported that the Δ^9^‐THC pharmacological profile at CB1 and CB2 receptors can range from partial/full agonist to antagonist depending on receptor density and coupling efficiency of downstream effectors. Thus, in certain tissues that have high expression of cannabinoid receptors (i.e., CB1R in substantia nigra and globus pallidus), agonist activity is predicted (Pertwee, 2005). While, several other reported investigations showed that Δ^9^‐THC behaved as antagonists in tissues that showed low expression level or coupling efficiency of both receptors, reviewed in Pertwee (1997, 2005, 2008). For example, using hippocampal cultures, the application of 100 nM of THC reversed the effect of 2‐AG on synaptic activity in a dose‐dependent manner (Kelley & Thayer, 2004). Additionally, it has been reported by Bayewitch et al. that THC behaved as a weak CB2R antagonist in Chinese hamster ovary cells (Bayewitch et al., 1996). Bhattacharyya et al. reported that in males the acute anxiety produced by THC is modulated in the right amygdala while processing fearful stimuli that are positively correlated with the extent of local CB1 receptors' availability (Bhattacharyya et al., 2017).

Moreover, CBD demonstrated a micromolar affinity range toward CBRs relative to Δ^9^‐THC, with *K*_*i*_ values ranging from 4350 to greater than 10,000 nM at CB1R and slightly higher affinity (*K_i_* ranging from 2399 to 10,000 nM) at CB2R. In terms of functional activity, multiple reports have shown that CBD antagonizes the agonist activity of CP 55,940 and R‐(+) WIN55212 and in some cases, it behaves as an inverse agonist at CB1R depending on the used concentration. At CB2R, CBD behaved as an inverse agonist with promising anti‐inflammatory properties in murine microglia and macrophages, reviewed in Pertwee (2004, 2008). The synthetic pathway of Δ^9^‐THC, CBD, and some other pCBs (i.e., cannabichromene [CBC]) share cannabigerol (CBG) as the main precursor that converts via specific synthase enzymes (e.g., Δ^9^‐THC synthase, CBD synthase, or CBC synthase) into various pCBs.

With regard to their oral effects, Gu et al. examined the influence of physiologically relevant doses of CBD, CBN, and THC (1.0 μg/ml) on the immune response induced by oral pathogens, *Porphyromonas gingivalis, Filifactor alocis*, and *Treponema denticola*. CBD, CBN, and THC exhibited the suppression of *P. gingivalis*‐induced cytokines while enhancing interleukin 10 (IL‐10, an anti‐inflammatory cytokine). Similar responses with the phytocannabinoids were also seen in *F. alocis‐* and *T. denticola*‐exposed human monocytes and human gingival keratinocytes. Interestingly, phytocannabinoid doses ≥5.0 μg/ml compromised cell viability, but efficiently inhibited the growth of *P. gingivalis* and *F. alocis*. Additionally, in a transient oral infection model, CBD was able to suppress *P. gingivalis*‐induced innate immune markers in wild‐type mice, but not in CB2R‐deficient mice (Gu et al., 2019). Although the phytocannabinoids can exert anti‐inflammatory effects, misuse of cannabis products could enhance PD and systemic diseases by promoting microbial dysbiosis via direct toxic effects on specific oral bacteria, suppression of the innate immune response to periodontal pathogens, and compromising oral cell vitality (Gu et al., 2019).

In animal studies, CBD has been shown to decrease nociception by inhibiting FAAH (fatty acid enzyme hydrolase that breaks down endocannabinoids) and consequently increasing AEA (endogenous cannabinoid). Therefore, AEA readily binds to CB1 and TRPV1 receptors, promoting analgesia while decreasing the nociception via TRPV1 receptors (Crivelaro do Nascimento et al., 2020; Peres et al., 2018). Moreover, CBD demonstrated the ability to enhance adenosine signaling by blocking its uptake and might be one of the mechanisms by which CBD modulates inflammation (Carrier et al., 2006). The NF‐κB is known to regulate the expression of genes associated with the pro‐inflammatory response (IL‐1β, tumor necrosis factor [TNF‐α], IL‐6) and cyclooxygenase‐2 (COX‐2). Interestingly, the number of cannabinoids and terpenes found in the hemp plant can inhibit NF‐κB directly or indirectly reducing inflammatory cytokines, thus attenuating inflammation (Morales et al., 2017; Simão da Silva et al., 2011; Kunnumakkara et al., 2018). Meanwhile, several cannabinoids (Muller et al., 2019) and flavonoids (Nakamura et al., 2016) found in the hemp plant activate transient receptor potential ankyrin 1 (TRPA1) (involved with cold hypersensitivity, acidic pH, hyperalgesia), while several cannabinoids activate the transient receptor potential vanilloid (TRPV) and rapidly desensitize the ion channel making them insensitive to mechanical, thermal, and chemical stimulation (Muller et al., 2019).

In MS patients, the mixture of THC: CBD oro‐mucosal spray has been used to treat pain and sleep disturbance in a randomized controlled trial in 2005. Cannabinoids adjunctive treatment demonstrated significant improvement in the pain and sleep disturbance in MS patients compared to the control group (Rog et al., 2005). Interestingly, a harmony in opioids and cannabinoid pathways exists; the antinociception measured in rats using high dose morphine or high dose THC was subjected to development of tolerance including receptor desensitization. Meanwhile, a low dose combination of both morphine and Δ^9^‐THC combination (0.75 mg/kg each, morphine subcutaneous and Δ^9^‐THC intraperitoneal) twice daily for 6.5 days circumvented the development of tolerance, while exhibiting antinociception (Smith et al., 2007). The use of phytocannabinoids in combination with opioids could potentially aid in the management of chronic pain in treating a variety of pain conditions.

## 6 SYNTHETIC CANNABINOIDS

Several studies have reported the beneficial effects of synthetic cannabinoids in treating multiple disease conditions. In particular, CB2R agonists demonstrated a promising ability to attenuate neuroinflammation in multiple disease models (Leleu‐Chavain et al., 2012). For instance, HU‐308 was discovered in 1999 and characterized as a selective CB2R agonist lacking CB1R activity (Hanuš et al., 1999). Application of HU‐308 in inflammatory models demonstrated anti‐inflammatory properties in vitro and in vivo studies (Gómez‐Gálvez et al., 2016; Taylor et al., 2015). Additionally, as reported in the literature, synthetic CB2R agonists are the most extensively studied ligands in the cannabinoids field (Aghazadeh Tabrizi et al., 2016). However, more recently, CB2R inverse agonists have emerged as a new potential therapeutic approach to treat neuroinflammation as reviewed by Lunn et al. (2008).

**Figure 2:**
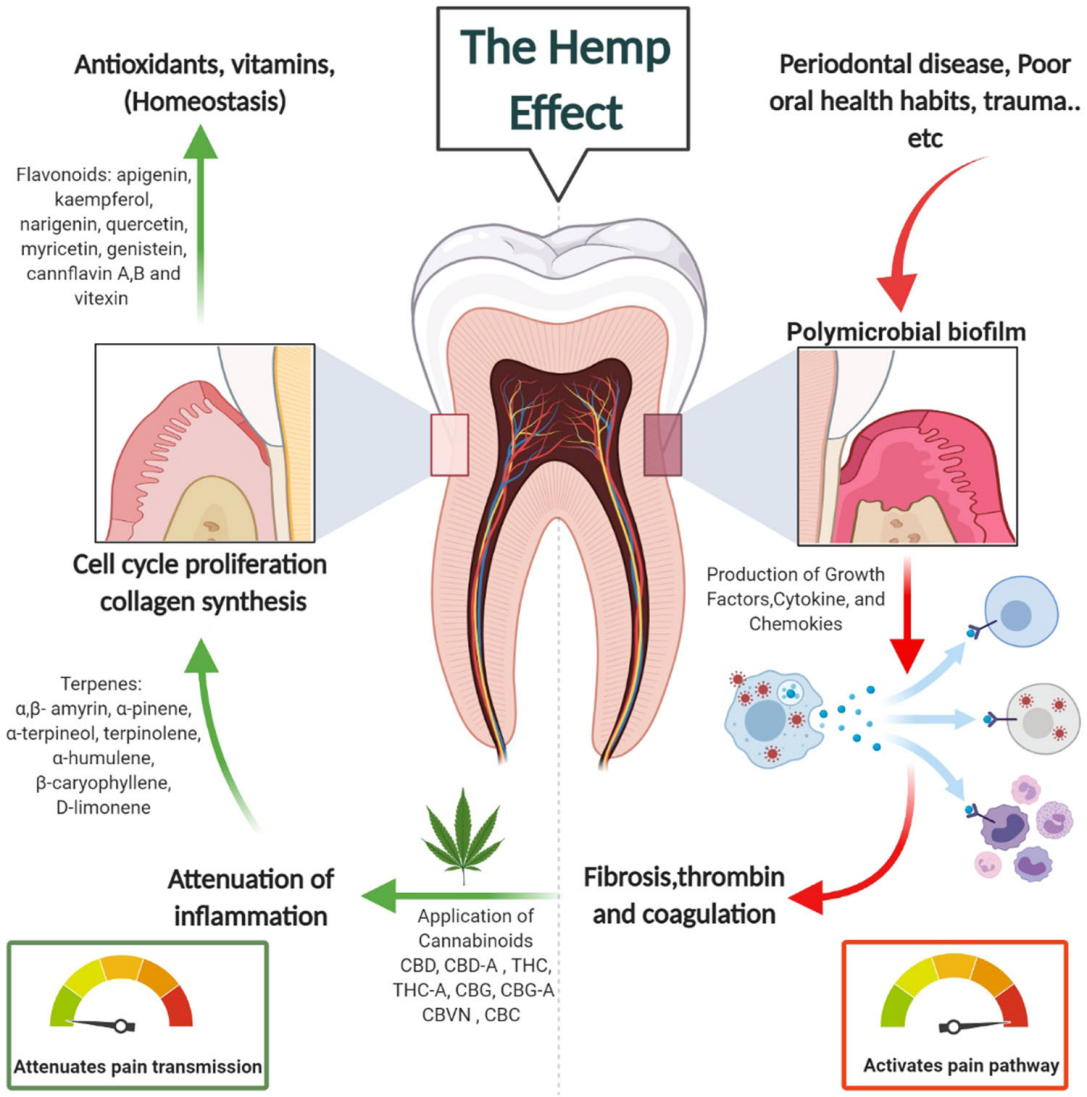
The immunomodulatory activity of phytocompounds found in hemp (cannabis) plant in dental‐associated inflammatory diseases including pulp pain. The diagram depicts the anti‐inflammatory activity of several natural compounds found in the hemp plant and the mode of impact to modulate and resolve pain and inflammation as an adjunct therapy. Several factors include poor oral hygiene and certain disease conditions (i.e., periodontitis) that initiate oral inflammation. After mechanical debridement of oral biofilms, the application of cannabis natural derivatives helps to attenuate the inflammatory process and controls inflammation. Created with BioRender.com

The pain management aspect of synthetic cannabinoids also shows great potential by alternating opioid and cannabinoid treatment, which could be therapeutically advantageous. The Δ^9^‐THC analog, HU‐210 enhances antinociceptive effects of morphine, when injected into the periaqueductal gray, preventing the development of tolerance, while enhancing morphine antinociception (Wilson et al., 2008). Additionally, selective modulation of the CB2R by AM1241 (agonist) caused the release of β‐endorphin from keratinocytes, decreasing pain by local neuronal μ‐opioid receptors (Ibrahim et al., 2005). These studies demonstrate antinociception through G‐protein‐coupled mechanisms by enhancing the opioid system leading to either improvement in opioid effects or by the local release of β‐endorphin by CB2R. In our previous studies, we have investigated the therapeutic potential of selective CB2R ligands to regulate chronic inflammation in PD. Our studies were mainly focused on investigating the immunomodulation properties of synthetic CB2R agonist HU‐308, and the CB2R inverse agonist SMM‐189 in stimulated primary human periodontal ligament fibroblasts (hPDLFs) in which synthetics could provide benefits in PD (Abidi et al., 2018). Moreover, other related findings showed that HU‐308 has prohomeostasis effects by inducing anti‐inflammatory and osteoprotective properties in oral tissues of rats with LPS‐induced PD (Ossola et al., 2016). These studies, along with others (Ossola et al., 2020; Zhang et al., 2020), highlight the important role of the ECS in oral and systemic health. Moreover, the cytokine and chemokine inhibition profiles of HU‐308 (agonist) and SMM‐189 (inverse agonist) were investigated in activated hPDLFs, and study results showed that CB2R ligands are viable candidates for the development of a new therapeutic intervention for PD (Figure 2) (Abidi et al., 2020).

## 7 CLINICAL PHARMACOKINETICS AND PHARMACODYNAMICS OF CANNABINOIDS

### 7.1 Pharmacokinetics

The plasma concentrations of the psychoactive components found in cannabis Δ^9^‐THC are subject to rapid decrease due to distribution into tissues and metabolism. The metabolic route for most cannabinoids occurs via the cytochrome P450 system and Δ^9^‐THC is primarily metabolized by CYP2C9, and with lower catalytic activity by CYP3A4 to an active metabolite is 11‐hydroxy‐Δ‐9‐tetrahydrocannabinol (11‐OH‐THC). Further oxidation of 11‐OH‐THC by CYP2C9 produces the inactive metabolite 11‐nor‐9‐carboxy‐Δ^9^‐tetrahydrocannabinol (THC‐COOH) (Bland et al., 2005; Watanabe et al., 2007). It is this inactive product (THC‐COOH) that serves as a biomarker for detection in clinical, forensic, and/or workplace testing (Karschner et al., 2012). The excretion or Phase II metabolism of THC is a bit more complex as the primary active metabolite 11‐OH‐THC is metabolized primarily by UDP‐glucuronosyltransferase enzymes (UGT1A9 and UGT1A10), while the inactive THC‐COOH is metabolized by UGT1A1 and UGT1A3 isoforms (Mazur et al., 2009). The THC‐COOH is more hydrophilic, which goes through renal clearance and hepatic elimination, accounting for about 20% of THC excreted as conjugated glucuronic acids and free THC hydroxylated metabolites. The majority of excreted THC is via feces, which accounts for 30%–65% (Wall et al., 1983).

Similar to THC, CBD is also metabolized by CYP2C9, CYP3A4 (Yamaori et al., 2012; Zendulka et al., 2016), and CYP2C19 (Stout & Cimino, 2014). The pharmacokinetics for CBD is complex and several metabolites may be generated, including the hydroxylation and oxidation at C‐7 of CBD. This can be further subjected to hydroxylation in the pentyl and propenyl groups that give rise to 1, 2, 3, 4, and 10‐hydroxy derivatives of CBD‐7‐oic acid (7‐COOH‐CBD) (Harvey & Mechoulam, 1990). The Phase II metabolism for CBD is glucuronidated by UGT1A9, UGT2B7, and UGT2B17 (Mazur et al., 2009), and similar to THC, CBD is excreted via feces and urine. Due to limited studies with other pCBs and their respective metabolites, probably similar CYP enzymes (CYP2C9, CYP3A4, and CYP2C19) with the addition of a few others may play a role in the breakdown and excretion of these pCBs. It is worth noting here that the “entourage effect” may be a consequence of the breakdown mechanism and circulating levels of metabolites in the blood affecting other cannabinoids. Interestingly, it has been reported that the psychotropic effects of THC are enhanced by CBD, which is due to the inhibition of CYP2C9 and CYP2C19 by CBD, which consequently decreases the clearance of THC (Yamaori et al., 2012). Meanwhile, the bioavailability and pharmacokinetics from oral–mucosal sprays and sublingual routes are similar (Millar et al., 2018), while data for plasma concentrations to achieve minimal effective dose and pharmacokinetics and bioavailability remain scarce (S. A. Millar et al., 2018, 2019).

## 8 PHARMACODYNAMICS

The impact of the cannabis products has also influenced cultural acceptance that includes smoking various forms of cannabis strains (e.g., Green crack, Sour diesel, etc.) due to presumed health benefits. Healthcare professionals, especially dentists may observe a higher incidence and progression of cannabis‐related oral health problems. Although the impact of hemp/CBD oil on oral health is unknown, marijuana is considered a risk factor for periodontal disease since it impairs immune response and compromises the healing of tissues (Tomar & Asma, 2000). The well‐known associated effects with marijuana usage are increased somnolence, appetite, and potent immunomodulatory effects that may be the underlying reason why cannabis users have more dental decay, poor oral hygiene, and greater plaque index than nonusers (Darling & Arendorf, 1992a; Shariff et al., 2017). Cannabis use has resulted in other documented intraoral conditions, including tongue carcinoma, gingival hyperplasia, xerostomia, uvulitis, and fiery red gingivitis (Almadori et al., 1990; Christie & Chesher, 1994; Rees, 1992; Rawal et al., 2012; Versteeg et al., 2008). Cessation of cannabis use along with nonsurgical and surgical therapies improved clinical and radiographic findings for a patient demonstrating a localized severe form of PD (Momen‐Heravi & Kang, 2017). Meanwhile, it is noteworthy that smoking (i.e., cigarettes) is a risk factor for developing periodontal disease; therefore, smoking cannabis products (ElSohly & ElSohly, 2007) (i.e., carbon monoxide, acetaldehyde, nitrosamines, and polycyclic aromatic hydrocarbons) poses a similar risk (Mayol et al., 2021). Although cannabis use is associated with poor oral health, cannabis use is not associated with other physical health problems in early midlife. However, tobacco use was associated with poor lung function, systemic inflammation, and metabolic health in early midlife (Meier et al., 2016).

Although cannabis has been associated with various oral conditions and adverse effects, it is generally well tolerated by most individuals. However, abuse of cannabis and/or products could result in an oral manifestation of diseases and possibly other conditions that are consistent with the well‐established role of the ECS and the cannabinoid receptors regulating immune response (Pacher & Mechoulam, 2011) modulation of T‐cells, B‐cells, production of inflammatory cytokines, chemokines, and cell migration (Croxford & Yamamura, 2005; Klein et al., 2003; Tanasescu & Constantinescu, 2010). The current shortcomings in our clinical understanding of marijuana exist largely due to incomplete knowledge of the complex biological activities of the phytocannabinoids and limited clinical studies. Other possible challenges are the limited scientific access to cannabis products and the lack of current consumer testing due to regulations and policies, resulting in limited data availability. For these reasons, the effects of many pCBs on the oral cavity are largely unknown, and what is available is focused on either Δ^9^‐THC (the psychotropic agent in marijuana) or CBD. It is very important to understand that the *C. sativa* plant contains many known pCBs, but little is known about their biological activity (Andre et al., 2016). In addition to cannabinoids, the cannabis plant also has terpenes, phenolic compounds including flavonoids that may modulate the overall health outcomes (Andre et al., 2016) and/or interact with other pCBs to produce opposing or enhancing (i.e., “entourage effect: *The whole plant is better than the sum of its parts*”) effects on the ECS (Laprairie et al., 2015; Pertwee, 2008; Russo, 2011) (Table 1).

**Table 1:** The effects and targets of components found in cannabis or hemp

	Effects	Target	References
*Phytocannabinoids*			
CBD	Analgesia, anxiolytic, antidepressant, anti‐inflammatory, antineoplastic	Agonist at 5‐HT1A, TRPV1, inhibits FAAH, modulates LOX‐5 and GABA‐A	Bakas et al. (2017); Davies (2011); De Petrocellis et al. (2011); Di Marzo et al. (2015); Fernández‐Ruiz et al. (2013); Hill et al. (2014); Kozela et al. (2016); Linge et al. (2016); Lu & Anderson (2017); Mahgoub et al. (2013); Morales et al. (2017)
CBG	Anti‐inflammatory, antiemetic, antineoplastic, stimulates appetite	Agonist TRPV1, 2, 4, inhibition of FAAH, antagonist at 5‐HT1A, COX‐2 inhibition	Borrelli et al. (2013, 2014); Cascio et al. (2010); Hill et al. (2014)
THC	Anti‐inflammatory, anxiolytic, analgesia	Agonist (partial) at CB1R, CB2R, PPAR‐γ, antagonist at 5‐HT3A	Appendino et al. (2011); De Petrocellis et al. (2011); Lowin & Straub (2015)
CBDA	Analgesia, anxiolytic, antidepressant, anti‐inflammatory, antineoplastic	Agonist at 5‐HT1A, TRPV1, COX‐2 inhibition	Bolognini et al. (2013); De Petrocellis et al. (2011); Takeda et al. (2008)
THCA	Anti‐inflammatory, neuroprotective	Weak binder at CB1R, CB2R, agonist of PPAR‐γ, TRPV1, inhibition (weak) of FAAH, COX 1,2	McPartland et al. (2017); Moreno‐Sanz (2016); Nadal et al. (2017)
*Terpenoids*			
Β‐Caryophyllene	Anti‐inflammatory, antibacterial, analgesia, antineoplastic	CB2R agonist, PPAR‐γ‐agonist	Alberti et al. (2017); Bahi et al. (2014); Fernandes et al. (2007); Gertsch et al. (2008a); Paula‐Freire et al. (2014); Rufino et al. (2015); Sharma et al. (2016)
Α‐Humulene	Anti‐inflammatory, antinociception, antineoplastic, antibacterial, insecticidal	Inhibition of AP‐1 and NF‐κB activation	Fernandes et al. (2007); Rogerio et al. (2009); Satsu et al. (2004)
Β‐Myrcene	Anti‐inflammatory, analgesia, sedative, muscle relaxant	Inhibits activation of NF‐κB	Guimarães et al. (2013); Rufino et al. (2015); de Cássia da Silveira e Sá et al. (2013)
A‐Pinene	Anti‐inflammatory, insect repellant, antifungal, bronchodilator	Inhibits activation of NF‐κB	Guimarães et al. (2013); D. S. Kim et al. (2015); McPartland (1997); Rufino et al. (2014)
A‐Terpineol	Promotes wound healing and anti‐inflammatory	Inhibition of COX‐2	Barreto et al. (2014) Khalil et al. (2004) de Cássia da Silveira e Sá et al. (2013)
*Flavonoids*			
Apigenin	Nephroprotective, antibacterial, antioxidant, antiviral, anxiolytic	Downregulates NF‐κB, inhibition of COX enzymes	Baptista et al. (2014); Hassan et al. (2017); Namratha et al. (2015); Pallauf et al. (2017); Panche et al. (2016); Weiskirchen (2016)
Genistein	Nephroprotective, reduction in liver fibrosis, phytoestrogen	Downregulates NF‐κB, inhibition of FAAH	Baptista et al. (2014); Namratha et al. (2015); Pallauf et al. (2017); Panche et al. (2016); Weiskirchen (2016)
Kaempferol	Antineoplastic, reduction of fatty lipids, antimicrobial,	Downregulates NF‐κB, inhibition of FAAH	Baptista et al. (2014); Calderón‐Montaño et al. (2011); Devi et al. (2015); Namratha et al. (2015); Pallauf et al. (2017); Panche et al. (2016)
Naringenin	Neuroprotective, hepatoprotective, antibacterial, preserves bone	Downregulates NF‐κB, inhibition COX‐2	Baptista et al. (2014); Chung et al. (2019); Pallauf et al. (2017); Panche et al. (2016); Tutunchi et al. (2020); Weiskirchen (2016)
Quercetin	Antihistamine, anti‐inflammatory, antimicrobial, antihypertensive, hepatoprotective	Downregulates NF‐κB, inhibition of 5‐LOX, and COX enzymes	Baptista et al. (2014); Lee et al. (2018); Maciel et al. (2013); Namratha et al. (2015); Pallauf et al. (2017); Panche et al. (2016); Serban et al. (2016); Weiskirchen (2016)

Abbreviations: 5 LOX, 5‐lipoxygenase; 5‐HT1A or 5HT3A, serotonin 1A or 3A receptor; AP‐1, activator protein 1; CBDA, cannabidiolic acid; CBD, cannabidiol; CBG, cannabigerol; COX 1,2, cyclooxygenase 1 or 2; FAAH, fatty acid amide hydrolase; GABA, γ‐aminobutyric acid; PPAR‐γ, peroxisome proliferator‐activated receptor‐gamma; THCA, tetrahydrocannabinolic acid; THC, tetrahydrocannabinol; TRPV, transient receptor potential cation channels

## 9 CANNABINOIDS AND DRUG INTERACTIONS

**Figure 3:**
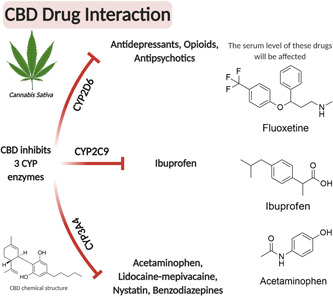
Interaction of cannabidiol (CBD) with various classes of traditional medicines that are metabolized by CYP enzymes. The expression levels of several CYP450 enzymes may be affected by CBD use (mainly through inhibition) leading to changes in serum levels of various drugs that indirectly affect the drug efficacy and treatment outcomes. Created with BioRender.com

As a dental or medical practitioner, several concerns may rise as the patient might use cannabis products along with other frequently used or prescribed medications in dentistry like nonsteroidal anti‐inflammatory agents (NSAIDs), local anesthetics, antimicrobial agents (antibiotics, antifungal, antiviral), corticosteroids, and antianxiety/sedative agents that could interact with cannabis or hemp extracts. For instance, the CYP3A4 is responsible for metabolism for almost a quarter of all drugs (Basheer & Kerem, 2015), and acetaminophen, lidocaine‐mepivacaine, nystatin, benzodiazepines are all mainly metabolized by CYP3A4. Additionally, CYP2D6 metabolizes many antidepressants, opioids, antipsychotics, while CYP2C9 is known to oxidize ibuprofen (Xie et al., 2012). Considering that CBD and THC both affect these enzymes, CBD in particular, by inhibition of CYP3A4, CYP2D6 (Yamaori et al., 2011), and CYP2C9 (Jiang et al., 2013) consequently affects the metabolism of many other drugs. Therefore, CBD and THC use could greatly affect the serum level of drugs that are metabolized by CYP3A4, CYP2D6, CYP2C9, including many dental drugs and other classes of drugs like SSRIs, opioids, antimicrobials, anxiolytics, and antipsychotics  (Qian et al., 2019) (Figure 3). Additionally, clinical trials with Epiodiolex report interactions with CYP1A2 (caffeine), CYP2B (bupropion), UGT1A9, UGT2B7, and clinically significant interactions with CYP2C8 and CYP2C9 (Phenytoin) when coadministered (Biosciences, 2018). The most significant adverse reaction (≥10% of an effect than placebo) seen in the patients receiving CBD included transaminase elevation (hepatocellular injury), while other significant effects included somnolence, decreased appetite, fatigue, vomiting, rash, insomnia, poor quality sleep, and infections (Biosciences, 2018).

Clinicians should be cautious when the patient is either taking cannabis or hemp‐based products as many metabolic pathways may be affected and there may be potential drug interactions that exist between cannabis‐hemp products and conventional medicine. There is a clear indication for the use of cannabinoids for the unmet clinical needs, but like most medications, cannabinoids can also produce adverse effects and toxicity. Several reviews have focused on the therapeutic and adverse effects of CBD with conclusions of CBD being generally safe but need more investigation (Machado Bergamaschi et al., 2011) and CBD's safety profile is better when compared to other antiepileptic and antipsychotics (Iffland & Grotenhermen, 2017). More recently, Huestis et al. (2019) reviewed CBD for its adverse effects and toxicity. In their review, the preclinical studies had shown adverse events negatively impacting cardiovascular, hepatocellular, hormonal changes, decreased fertility, alterations of in vitro cell viability, and P‐glycoprotein effects; however, these effects occurred when doses exceeded 200 mg/kg/day, which is far above the 50 mg/kg/day suggested in antiepileptic clinical studies. It is important to note that the clinical trial data has reported only a few incidences of cardiovascular and reproductive effects. Cannabis and hemp‐based products seem to be well tolerated by the body; however, it is not risk‐free but may be relatively safer than some prescription and/or narcotic drugs (Foster et al., 2019). Additionally, the significantly high doses of CBD or the route of administration (IP or IV) can also alter overall cannabinoid content; therefore, the chances of adverse effects can increase that need to be monitored with hemp or CBD‐rich oils.

## 10 LEGAL STATUS AND CANNABINOID PRODUCTS

Under current federal law (United States), possession of cannabis is illegal, where cannabis is categorized under schedule I drug status. However, many states have allowed the use of cannabis (marijuana) for medical and recreational purposes, while also decriminalizing it (MARIJUANA‐OVERVIEW, 2017). In the Agriculture Improvement Act or the Farm Bill of 2018, FDA explicitly preserves the authority over industrial hemp or “hemp” defined as *C. sativa L* with Δ^9^‐THC (psychotropic) content to be no more than 0.3% on a dry weight basis, while separating marijuana as an independent identity, removing hemp from being a control substance. Hemp is being marketed for various CBD‐rich or hemp oil‐rich products (oral oil drops, capsules, topical lotions, creams, and teas) that are seeing considerable commercial growth. According to Forbes magazine, it is estimated that US sales of hemp/CBD products will exceed $20 billion by 2024. The current CBD/hemp oil industry boasts the effects of CBD to improve a variety of conditions like pain (Good et al., 2019), migraines, and other inflammatory conditions (Burstein, 2015). Moreover, in recent findings, CBD oils may aid in reducing opioid addiction. A clinical study reported that among patients using opioids for pain management, CBD hemp extract significantly provided chronic pain relief along with the reduction of opioid use and improved sleep quality among CBD‐treated patients (Capano et al., 2020). The hemp/CBD oils affect the body by modulating receptor systems specifically targeting the ECS (Croxford & Yamamura, 2005; Jean‐Gilles et al., 2015; Klein et al., 2003). The main ingredients found in hemp oils are CBD along with other cannabinoids, terpenoids, and polyphenolics (flavonoids) that exhibit a synergistic effect on the body termed the “Entourage Effect.”

## 11 EFFICACY AND SAFETY OF CANNABINOIDS

Many conditions or diseases are considered multifactorial, and individuals who prefer holistic therapy tend to be interested in natural products. Therefore, the use of full‐spectrum hemp (whole extract) is gaining the interest of many, which provide a multitargeted treatment, and could potentially have therapeutic advantages. In dental plaque samples, cannabinoid‐infused mouthwash (CBD, CBG) was compared to the gold standard 0.2% chlorhexidine digluconate for inhibition of total cultured bacteria (aerobes). The cannabinoids infused mouthwash were equally as effective in decreasing bacterial load and show a promise for oral healthcare use (Vasudevan & Stahl, 2020). Alex Capano et al. investigated the impact of full hemp extract CBD on opioid use and quality of life among chronic pain patients. The concentrations of 15, 30, and 60 mg of CBD were used daily for 8 weeks with very limited side effects and only a few patients were excluded from the study due to adverse effects.

The CBD‐rich hemp oil components (may vary based on vendor) other than CBD are usually at small concentrations and are unlikely to cause any severe reactions but do play an important role in the modulation of host immune responses. It is interesting to note that terpenoids like β‐caryophyllene that exist in the *C. sativa* plant have been reported to exist in various other plants and are commonly found in foods like oregano, African black pepper, ginseng, and so forth (Gertsch et al., 2008). β‐Caryophyllene has been shown to exert analgesic effects in inflammatory and neuropathic animal pain models (Katsuyama et al., 2013; Klauke et al., 2014). A limited examination of the essential oils from a hemp variety exhibited positive relaxation and anxiolytic effects in the patient population, while it also improved brain wave activity, autonomic nervous system response, and enhanced mood states. The components attributed to these effects include α‐pinene, terpinolene, α‐humulene, witincludephyllene, and myrcene being main components (Gulluni et al., 2018). A list (Russo, 2011) of cannabinoids: CBD, cannabidiolic acid (CBD‐A), THC, tetrahydrocannabinolic acid (THC‐A), CBG, cannabigerolic acid (CBG‐A), tetrahydrocanncannabigerol), CBC terpiods: α,β‐ amyrin, α‐pinene, α‐terpineol, terpinolene, α‐humulene, β‐caryophyllene, d‐limonene, and flavonoids: apigenin, kaempferol, naringenin, quercetin, myricetin, genistein, cannflavin A, B and vitexin can all be found in the cannabis/hemp plant with properties, including but not limited to antinociception, neuroprotection, anti‐inflammatory, anxiolytic, antimicrobial, osteoprotective, antihypertensive, and improvement of wound healing and sleep (Nahler, 2018). Moreover, the difference between CBD oil, hemp oil, or cannabis oil is the relative amount of CBD to other phytocannabinoids present in the extract, which can range from small to large portions and are not usually identified from batch to batch. Due to this reason, the commercial content of hemp with regard to CBD and Δ^9^‐THC are often incorrectly labeled (Vandrey et al., 2015; Welty et al., 2019).

Additionally, the studies with CBD approved by the FDA for the treatment of seizures in Lennox–Gastaut Syndrome and Dravet Syndrome (Franco & Perucca, 2019) provide better evidence of CBD safety highlighting some risks (discussed later) and a potential for liver injury. Therefore, clinicians interested in recommending hemp/CBD oil for pain, anxiety, or sleep should make sure that the patient does suffer from hepatic diseases. It is worth noting that CBD has been given in significantly high dosing for patients with bipolar, manic episodes, antipsychotic, epilepsy, anticonvulsive ranging from 300 m/day for 6 months, and up to 4 weeks with dosing 1200–1500 mg/day with reported side effects, which include somnolence, decreased appetite, and diarrhea (Cunha et al., 1980; Machado Bergamaschi et al., 2011; A. Zuardi et al., 2010; A. W. Zuardi et al., 1995).

The rise of the opioid abuse epidemic and healthcare challenges to manage pain has resulted in the search for alternative pain therapy. Cannabis or CBD/hemp‐rich extract provides a great alternative therapy for the reduction of acute and chronic pain that may also aid in dental pain management. About 9% of the total opioid prescriber population are dentists, of which 45% of opioid prescriptions in the United States are written by dentists (McCauley et al., 2016). While the overall trends of prescribing opioids are decreasing in the United States, there is evidence of increasing rates in dental settings (Gupta et al., 2018; Guy et al., 2017). This issue of opioid prescribing in dentistry is particularly problematic in the United States as the prescriptions written for opioids by US dentists are 37 times greater than those of dentists in England (Suda et al., 2019).

Preclinical studies have demonstrated that cannabinoids (i.e., cannabis plants) enhance the body's response to pain by binding with the endocannabinoid receptors (Vučković et al., 2018). In 2017, the National Academy of Sciences (NAS) assembled an Expert Review Committee in which they concluded: “there is substantial evidence that cannabis is an effective treatment for chronic pain in adults” (National Academies of Sciences, 2017). Additionally, a systemic review on randomized clinical trials (RCTs) conducted for cannabinoids for medical use provided moderate‐quality evidence to support the use of cannabinoids for the treatment of chronic pain and spasticity, while association in short‐term adverse effects was found (Whiting et al., 2015). Moreover, the National Academies of Sciences (2017) provided evidence that cannabis is modest in treating pain, discrediting it as a “miracle drug,” and debunking that individuals use it as an excuse to “get high or stoned.” The National Institutes of Health (NIH) has awarded a grant for a prospective cohort study expected to shed light on the understanding of how medical cannabis use affects opioid analgesic use over time, with specific attention to THC/CBD content, adverse events, and other health outcomes (ClinicalTrials.gov Identifier: NCT03268551). For a short excellent review on cannabis/substitute for opiates in the management of chronic pain please read (Carlini, 2018).

There is a growing body of evidence that cannabis can result in synergism and/or additive effects in combination with opioids for the resolution of pain. The indirect stimulation of opioid receptors by CB2R mediated in primary afferent pathways (Ibrahim et al., 2005, 2006) the colocalization of CB1R and μ‐opioid receptor in the spinal cord at the first synaptic contact for peripheral nociceptive afferent neurons (Hohmann et al., 1999; Salio et al., 2001) support the synergistic enhancement of opioid analgesia along with the cannabinoids direct analgesic effects. A systemic review on opioid‐sparing effects of cannabinoids concluded that findings from clinical studies are inconsistent due to limitations (i.e., lack of placebo control). However, most of the preclinical studies found synergistic effects in which the meta‐analyses found the dose of morphine and codeine required to produce the same analgesic effects were lowered by 3.6 and 9.5, respectively, when coadministered with delta‐9‐THC (Nielsen et al., 2017). Therefore, cannabinoids may provide enormous clinical relevance to practitioners that are looking for effective pain treatment by utilizing opioid‐sparing medications to lower opioid doses and dependence thereby reducing opioid‐related mortality. Urits et al. reviewed certain cannabinoids and cannabinoid extracts investigating both clinical and preclinical evidence that supported cannabinoid pharmacotherapy for pain (Urits et al., 2019). The CBD dosing in clinical studies has ranged from 5 to 800 mg/day (Fasinu et al., 2016) and the concentrations to determine low or high doses for pain management for phytocannabinoids exhibit U‐shaped dose responses (Katsidoni et al., 2013; Kwiatkowska et al., 2004). For instance, studies examining anxiolytic effects of CBD report that a low dose of 3 or 10 mg/kg show anxiolytic and antidepressant effects, while doses of greater than 10–30 mg/kg in mice after 15–30 days did not show the same anxiolytic effects (Schiavon et al., 2016).

Due to the current research barriers and the increase in current cannabis products, the scientific capacity to assess or study many of the currently used cannabis products becomes very difficult. While products, such as nabiximols (an oral spray used to alleviate neuropathic pain and spasticity), are commercialized in 30 countries, including Europe and Canada, have yet to get approval by the Food and Drug Administration (FDA) in the United States. The FDA has shown a commitment to advancing hemp products and is making appropriate additional regulatory pathways to hemp products such as those containing CBD with the intent to protect patients and public health (Hemp Production and the 2018 Farm Bill, in Agriculture, Nutrition, and Forestry, 2019). Therefore, there is an urgent need to increase the awareness of healthcare professionals, especially dental professionals to understand the potential impact of hemp or cannabis products on the quality of life with possible detriment or beneficial outcomes. CBD is also being tested under a dental setting to manage pain after simple tooth extraction (acute inflammatory pain) as a means of alternative pain management for dental patients (ClinicalTrials.gov Identifier: NCT04271917). Additionally, safety concerns do exist with CBD and preclinical studies exhibit potent induction and inhibition of cytochrome P450 (CYP450) (Jiang et al., 2011) (e.g., CYP2C, CYP2D6, and CYP3A isoforms), and limiting dosing of CBD to 30–120 mg/day might be more appropriate for clinicians to reduce drug–drug interactions or complications.

## 12 CHALLENGES AND CONSIDERATION FOR UTILIZING CANNABINOID PRODUCTS

The Clinical Guide of CBD and Hemp oil (VanDolah et al., 2019) states that physicians who want to examine CBD and hemp oils need to find the highest‐quality product as variations occur in commercial products from the declared amount (Pavlovic et al., 2018). The recommendation of the isolation method includes products extracted by carbon dioxide with no solvents and certified by the US Department of Agriculture with testing performed for pesticides/herbicides. Since hemp seed oil does not contain any of the phytocannabinoids or terpenoids, be aware that the oil being used has nutritious omega‐3 fatty acids but lacks cannabinoid content. Additionally, pure CBD oils have been studied much more rigorously but the full spectrum of cannabis products may be beneficial but have not been subjected to more research concerning their safety and efficacy (VanDolah et al., 2019).

Unfortunately, many of the studies with hemp extracts reference only phytocannabinoids and do not address the composition of other phytocompounds. There is also a discrepancy between human and animal studies with regard to the effects of cannabis extracts, in part due to a higher dosage of 8–40 mg/kg in animals compared to human dosage is about 0.25 mg/kg (Katona et al., 2005; Zgair et al., 2017). Additionally, various experiments do not necessarily favor either extracts or pure cannabinoids like CBD/THC. Therefore, the question of a high number of terpenes, flavonoids, and/or CBD/THC ratio would improve specific outcomes remains unknown. The current shortcomings in our understanding of the clinical effects attributed to CBD‐rich or hemp oil extracts are largely due to inconsistency of stain, condition of growth, harvest factors, drying, and extraction methods, which have significantly limited clinical publications. Increased literary and scientific support for CBD‐rich hemp extracts is on a rise, which should help develop a more coherent and clinically relevant regimen for the restoration of host homeostasis by the management of pain, inflammation, immune responses, and anxiety.

## 13 CONCLUSION

There is significant interest in *C. sativa* due to the growing medical and public interest in its use for multiple conditions. The approval of CBD for the treatment of rare epileptic conditions for which the US Drug Enforcement Administration (DEA) changed its status to Schedule V (low abuse potential) has opened CBD's research potential. There is evidence that shows cannabinoids are clinically relevant in reducing pain symptoms and are a potential avenue for pharmacotherapy as opioid abuse and related deaths remain high in the United States. Due to the heavy reliance on opioids to manage pain following surgeries, the development of nonaddictive analgesics is highly desirable, and a target of interest for the NIH. The pharmacological effects of pure CBD have been studied extensively with several adverse effects seen in both preclinical and clinical settings. Meanwhile, cannabis extracts and hemp products have gained much attention but can vary significantly and lack empirical studies. There are several CBD products (unapproved) sold across the globe without any standardization of CBD or other constituents that claim unproven health effects. Although cannabis or cannabis‐related products exhibit anti‐inflammatory properties, it is unclear how well hemp/CBD products can replace traditional pain management, or if these products will work in tandem with existing therapies to enhance the effects and reduce the duration of analgesic care. At this point, CBD‐rich or hemp oils are considered safe, but CBD's reported side effects and drug interactions are not negligible and must be considered before therapeutic recommendation. The potential of medical use of cannabis or hemp/CBD still has great interest but its acceptability with practitioners and patients alike is rather low due to incomplete knowledge or bias, which poses several challenges to uptake. As a healthcare professional, it is important to understand that just like common medications are not suitable for every individual, neither is CBD‐rich or hemp product. However, since CBD's efficacy for seizures has been proven, it is likely physicians who are comfortable with CBD usage will recommend off‐label use for other conditions. Given the healthcare challenges to manage acute and chronic pain, we are currently investigating CBD‐rich hemp extract therapy in patient populations to be used for the reduction of acute and chronic dental pain, while examining specific phytocannabinoids and their respective effects in primary cell cultures to characterize them for a clinical indication and health benefits. Finally, the current shortcomings in understanding the benefits of CBD‐rich hemp oils have limitations due to pharmacological and clinical effects not being predictable and profiles of marketed products varying greatly in phytocompounds.

## AUTHOR CONTRIBUTIONS

Ammaar H. Abidi and Sahar S. Alghamdi drafted the manuscript, contributed to conception and design, contributed to acquisition, analysis, and interpretation, and critically revised the manuscript. Karen Derefinko drafted the manuscript, contributed to acquisition, analysis, and interpretation, and critically revised the manuscript.

## CONFLICTS OF INTEREST

The authors declare no conflicts of interest.

14

## Data Availability

The article submitted is a review article and no data availability statement is required.
